# The calcium-sensing receptor in inflammation: Recent updates

**DOI:** 10.3389/fphys.2022.1059369

**Published:** 2022-11-18

**Authors:** Luca Iamartino, Maria Luisa Brandi

**Affiliations:** ^1^ Department of Experimental Clinical and Biomedical Sciences “Mario Serio”, University of Florence, Florence, Italy; ^2^ F.I.R.M.O. (Italian Foundation for the Research on Bone Diseases), Florence, Italy

**Keywords:** calcium-sensing receptor (CaSR), inflammation, calcilytics, calcium, immune response, SARS-COVID-19

## Abstract

The Calcium-Sensing Receptor (CaSR) is a member of the class C of G-proteins coupled receptors (GPCRs), it plays a pivotal role in calcium homeostasis by directly controlling calcium excretion in the kidneys and indirectly by regulating parathyroid hormone (PTH) release from the parathyroid glands. The CaSR is found to be ubiquitously expressed in the body, playing a plethora of additional functions spanning from fluid secretion, insulin release, neuronal development, vessel tone to cell proliferation and apoptosis, to name but a few. The present review aims to elucidate and clarify the emerging regulatory effects that the CaSR plays in inflammation in several tissues, where it mostly promotes pro-inflammatory responses, with the exception of the large intestine, where contradictory roles have been recently reported. The CaSR has been found to be expressed even in immune cells, where it stimulates immune response and chemokinesis. On the other hand, CaSR expression seems to be boosted under inflammatory stimulus, in particular, by pro-inflammatory cytokines. Because of this, the CaSR has been addressed as a key factor responsible for hypocalcemia and low levels of PTH that are commonly found in critically ill patients under sepsis or after burn injury. Moreover, the CaSR has been found to be implicated in autoimmune-hypoparathyroidism, recently found also in patients treated with immune-checkpoint inhibitors. Given the tight bound between the CaSR, calcium and vitamin D metabolism, we also speculate about their roles in the pathogenesis of severe acute respiratory syndrome coronavirus-19 (SARS-COVID-19) infection and their impact on patients’ prognosis. We will further explore the therapeutic potential of pharmacological targeting of the CaSR for the treatment and management of aberrant inflammatory responses.

## Introduction

The calcium ion, Ca^2+^, is a ubiquitous molecule implicated in many physiological processes that include neuronal transmission, muscle contraction, bone mineralization, immune response, and hormone secretion, among others. At the cellular level, Ca^2+^ regulates cell-cell adhesion and, as an intracellular messenger, it controls gene transcription, cell proliferation, and apoptosis (Berridge et al., 2003; Clapham, 2007; Bagur and Hajnóczky, 2017). Due to its pleiotropic roles, calcium imbalance can lead to multiorgan disfunctions; therefore, the human body is equipped with a sophisticated system that guarantees calcium homeostasis.

A key regulator for serum calcium balance is the CaSR, a GPCR that directly controls calcium reabsorption in the kidneys and regulates PTH release from the parathyroid glands. In turn, PTH controls calcium resorption in the bones, calcium excretion in the kidneys and, by promoting renal synthesis of the 1,25-dihydroxyvitamin D3 (the active form of vitamin D), calcium absorption in the intestine (Hofer and Brown, 2003; Hannan et al., 2018).

The CaSR is a ubiquitously expressed receptor able to bind not only Ca^2+^, but a plethora of other ligands, that confers ligand-biased signaling (Conigrave and Ward, 2013). Depending on the type of ligand and where the receptor is expressed, the CaSR is able to exert multiple physiological roles, such as neuronal development, vessel tone, insulin secretion, bowel fluid absorption, and many others. At the cellular level, the CaSR is able to regulate cell transcription, proliferation, differentiation, and apoptosis. Given this multifunctionality, the CaSR also seems to play an important role in cancer development and inflammatory response (Brennan et al., 2013; Tennakoon et al., 2016; Hannan et al., 2018; Iamartino et al., 2018).

Depending on the tissue where the CaSR is expressed, the CaSR can either promote cancer development, thus behaving as an oncogene, such as in gastric, breast, prostate, and renal cancer, or as a tumor suppressor, such as in colorectal, endometrial, parathyroid cancer, and neuroblastoma (Tennakoon et al., 2016).

This yin-yang role in cancer is also observed in inflammation. Multiple studies, especially in the last decade, have reported that the CaSR seems to promote inflammation in several tissues, while, mainly in the intestine, there are contradicting reports (Iamartino et al., 2018; Elajnaf et al., 2019; Iamartino et al., 2020). With the present review, we aim to give recent updates on the impact of the CaSR in immune response and tissue inflammation. Given the strong relationship between the CaSR, vitamin D and calcium, and the impact of the latter in viral infection and immune modulation, we will speculate about the possible implication of the CaSR-vitamin D-calcium asset on the pathophysiology of SARS-COVID-19 infections and on the prognosis of infected patients.

## General aspects of the calcium-sensing receptor

### Calcium-sensing receptor gene, protein structure and signaling

The *CaSR* gene is located in the 3q13.3-21 of the human genome (Janicic et al., 1995) and is composed of 8 exons; of those, two alternative exons, 1A and 1B, are located within the 5′-untranslated region (5′UTR) and are transcribed from two distinct promoters. Both 1A and 1B splice to exons 2–7 that translate the whole protein, which is comprised of 1,078 amino acids (Garrett et al., 1995; Chikatsu et al., 2000; Hendy and Canaff, 2016b).

Similar to the other receptors of the family C of GPCRs (i.e., metabotropic glutamate receptors (mGluRs), γ-aminobutyric acid_B_ (GABA_B_) receptors, and some taste receptors), the CaSR is comprised of a large extracellular domain (ECD) with a bilobed structure, termed Venus Flytrap domain (VFT), deputed to ligand binding. The CaSR anchors to the plasma membrane with a 7 α-helices transmembrane domain (7TM), which is connected to the ECD through a cysteine reach domain. With its intracellular C-terminal domain, the CaSR is able to interact with the heterotrimeric G proteins and with β-arrestin, thereby activating diversified signaling cascades. By binding α_i/o_, α_q/11_ and α_12/13_, the CaSR is able to inhibit adenylate cyclase and cAMP synthesis, activate the MAPK cascade through extracellular-signal regulated kinase (ERK) phosphorylation, promote intracellular calcium release by activating the phospholipase C (PLC)-inositol 1,4,5-triphosphate (IP_3_) cascade, and induce membrane ruffling. Furthermore, the CaSR, by binding β-arrestin, is able to modulate its own endocytosis and ERK phosphorylation (Hofer and Brown, 2001; Hofer and Brown, 2003; Brauner-Osborne et al., 2006; Brennan et al., 2013; Conigrave and Ward, 2013; Hannan et al., 2018).

The CaSR was first identified in the parathyroid glands as a “calcium-sensing” receptor (Brown et al., 1993); however, later studies reported it to be sensitive also to other ions, such as Mg^2+^ and Gd^3+^, and to a plethora of other molecules that stimulate its activity, including aminoglycoside antibiotics (e.g., neomycin), polypeptides (i.e., poly-l-arginine, poly-l-lysine and amyloid β peptides), polyamines (i.e., spermine, spermidine and putrescine), glutamyl dipeptides, and some amino acids, such as phenylalanine and tryptophan (Hofer and Brown, 2003; Tennakoon et al., 2016; Iamartino et al., 2018). The broad diffusion and variety of these molecules allow systemic activation of the CaSR in different tissues, conferring a multiplicity of cellular responses.

### Physiological roles of the calcium-sensing receptor

The best described physiological role of the CaSR is its capability to control serum calcium balance [please see the following refs (Hofer and Brown, 2001; Brennan et al., 2013; Hannan et al., 2018)]. The importance of the CaSR for calcium homeostasis is the fact that CaSR mutations are associated with inherited diseases characterized by calcium and PTH disbalance. Hypercalcemic diseases can derive from loss-of-function CaSR mutations, such as type 1 familial hypocalciuric hypercalcemia (FHH1) and neonatal severe hyperparathyroidism (NSHPT) (Chattopadhyay and Brown, 2006; Hannan et al., 2012; Hannan and Thakker, 2013; Hannan et al., 2016), while gain-of-function CaSR mutations are associated with hypocalcemic disorders, such as type 1 autosomal dominant hypocalcemia (ADH1) and type 5 Bartter syndrome (Vargas-Poussou et al., 2002; Chattopadhyay and Brown, 2006; Hannan and Thakker, 2013; Hannan et al., 2016; Hannan et al., 2018).

The impairment of CaSR expression/functionality is the main culprit of primary and secondary hyperparathyroidism. The former is caused by the development of hyperplasia or adenoma of generally one of the four parathyroid glands, while the latter results as a complication of chronic kidney disease (CKD), where hyperphosphatemia, caused by kidney failure, induces the aberrant enlargement of the parathyroid glands. In both conditions, the hyperplastic parathyroid glands fail to regulate serum Ca^2+^ homeostasis, leading to the increase of PTH secretion. While primary hyperparathyroidism tends to be asymptomatic, the late-stages of secondary hyperparathyroidism are characterized by concomitant hyperphosphatemia and hypercalcemia that are associated to life-threatening vascular calcifications (Fraser, 2009; Cunningham et al., 2011; Naveh-Many and Volovelsky, 2020). While primary hyperparathyroidism is mostly treated surgically, secondary hyperparathyroidism can be treated pharmacologically, in particular by stimulating the CaSR either through the FDA-approved cinacalcet, a synthetic positive allosteric CaSR modulator, or with the newly clinically approved etelcalcetide, a recently developed synthetic CaSR agonist (Nemeth et al., 2004; Fraser, 2009; Cunningham et al., 2011; Hamano et al., 2017).

In addition to its “calcitropic” functions, the CaSR plays other physiological roles in the body, comprehensively reviewed by S. Brennan et al. and Hannan et al. (2018) (Brennan et al., 2013). Moreover, the CaSR has been found to be a multifunctional factor in cancer, as reviewed in detail by Tennakoon et al. (2016).

**FIGURE 1:**
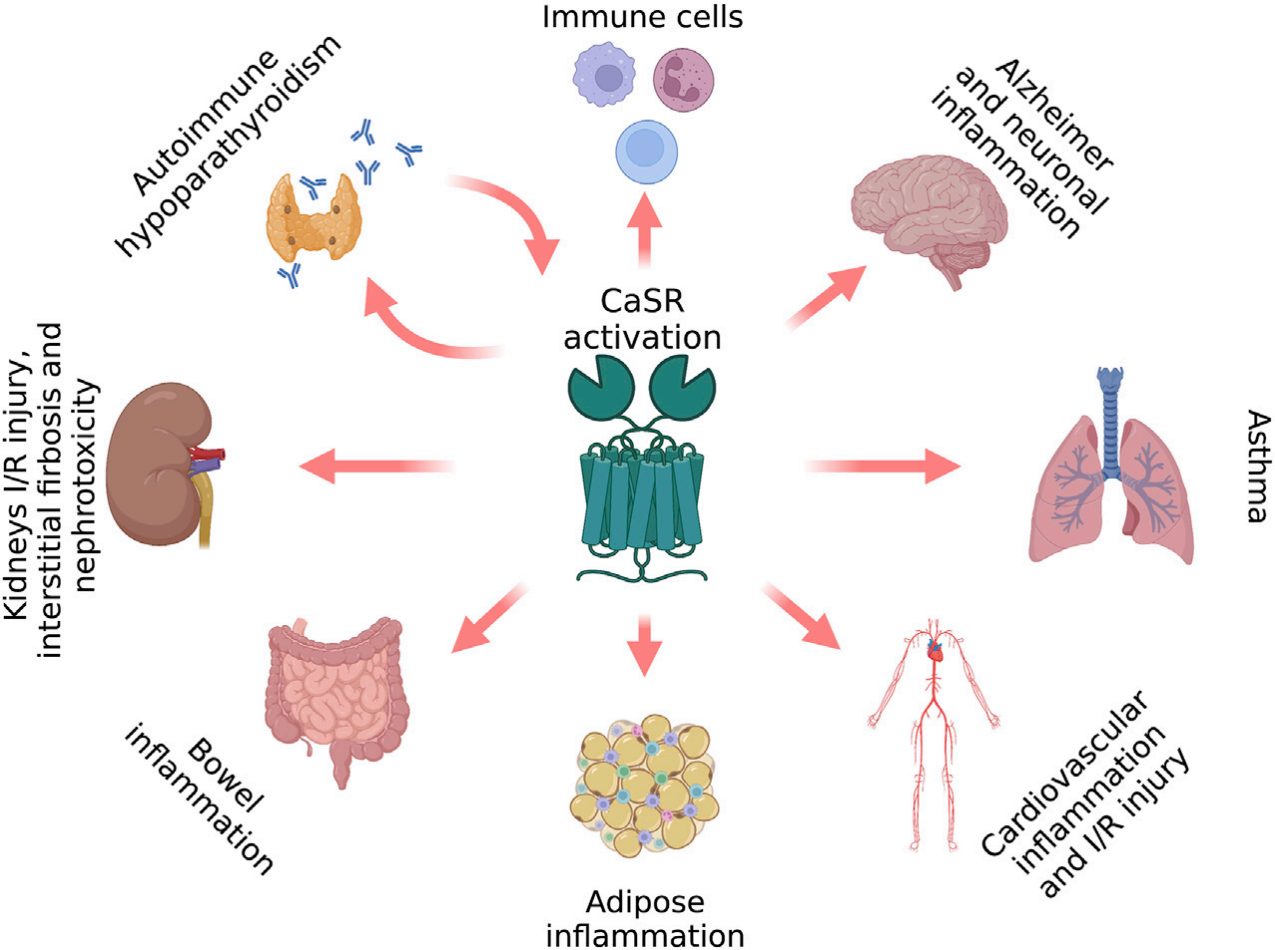
CaSR-induced inflammatory hallmarks (figure drawn with Biorender.com, accessed on 27 October 2022).

Because calcium plays pivotal roles in inflammation, its receptor, the CaSR, is an important mediator of the inflammatory processes. Compelling evidence has demonstrated that the CaSR mediates inflammation in various tissues and immune cells (Figure 1), where it mediates their activity and chemokinesis.

## Cross-talk between the calcium-sensing receptor and inflammation

Numerous studies have reported a correlation between PTH/calcium perturbations and inflammation (Hendy and Canaff, 2016a). In rheumatoid arthritis, PTH secretion was found to be impaired and its level inversely correlated with inflammation (Ekenstam et al., 1990). Other clinical studies have reported that hypocalcemia is frequently observed in critically ill patients and in case of severe burn injury (Gary, 1992; Klein et al., 1997; Lind et al., 2000; Steele et al., 2013). Even ill horses suffering from enterocolitis manifest hypocalcemia (Toribio et al., 2001).

Cytokines are the principal mediators of inflammatory signals and, upon immune reaction, their levels increase to sustain a proper immune response. Evidence of the impact of the cytokines on calcium metabolism came from *in vitro* observations where IL-6 was found to suppress PTH secretion in bovine parathyroid glands (Carlstedt et al., 1999) as well as IL-1β, which was found to further upregulate CaSR mRNA in both bovine (Nielsen et al., 1997) and equine parathyroid glands (Toribio et al., 2003). The CaSR, therefore, has been hypothesized to be responsible for the calcium/PTH disbalance in inflammation. Several *in vivo* studies have confirmed this assumption, reporting that pro-inflammatory cytokines were able to up-regulate the CaSR. In sheep models of burn injury, the high level of circulating pro-inflammatory cytokines up-regulated CaSR expression in the parathyroid glands (Murphey et al., 2000). Canaff et al. (2008) showed that rats receiving intraperitoneal injection of IL-1β showed an up-regulation of CaSR mRNA and protein in parathyroid glands, thyroid, and kidneys, while serum calcium, PTH, and 1,25-dihydroxyvitamin D3 were reduced (Canaff and Hendy, 2005). The same effects were also observed after intraperitoneal injection of IL-6 (Canaff et al., 2008). Because of its ability to lower serum calcium content and inhibit parathyroid cell proliferation (Miller et al., 2012; Brennan et al., 2013), it was hypothesized that CaSR up-regulation could explain the hypocalcemia and hypoparathyroidism found in patients with severe burn injuries and the reduction of serum calcium content in ill patients (Gary, 1992; Klein et al., 1997; Lind et al., 2000; Steele et al., 2013).

**FIGURE 2:**
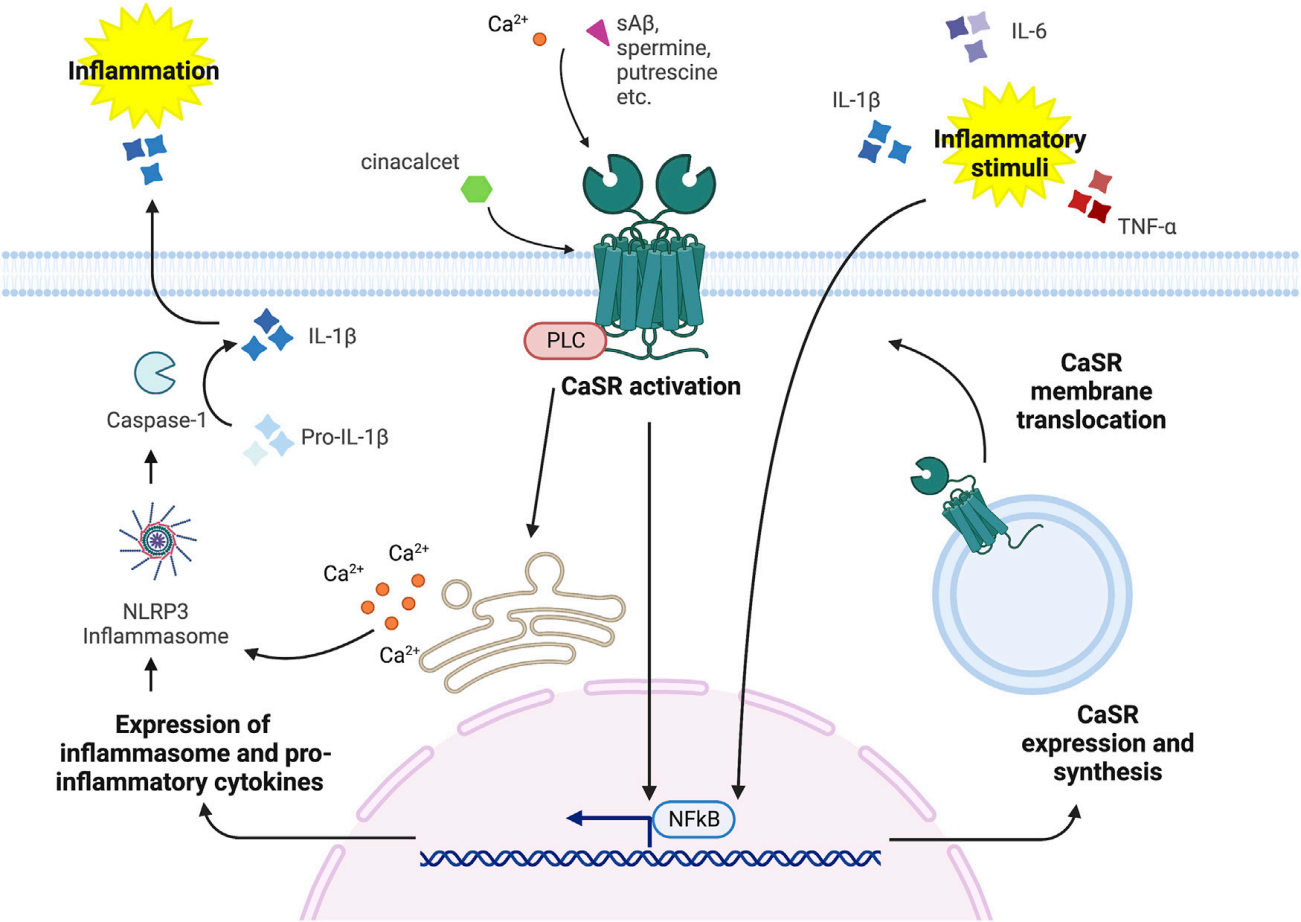
CaSR-Inflammation signaling cross-talk (figure drawn with Biorender.com, accessed on 27 October 2022). CaSR stimulation *via* orthosteric ligands, such as Ca^2+^, spermine, spermidine, putrescine, etc. and allosteric modulators, such as cinacalcet and NPS R-568, induces the activation of the NLRP3 inflammasome and the NF-κB pathway that promote inflammatory stimuli by releasing pro-inflammatory cytokines. In turn, inflammatory stimuli, i.e., IL-6, TNF-α and IL-1β, can induce CaSR expression and synthesis.

The induction of CaSR expression from pro-inflammatory cytokines (as depicted in Figure 2) is most likely due to specific transcription regulatory elements located in the CaSR locus, which are activated by pro-inflammatory stimuli. Canaff and Hendy (2005) found that IL-1β induces CaSR transcription through the NF-κB pathway that targets κB-specific transcription response elements in both CaSR promoters. Other pro-inflammatory cytokines are also able to promote CaSR expression, such as TNF-α *via* NF-κB signaling (Fetahu et al., 2014) and IL-6, by activating the JAC-STAT pathway that targets the STAT1/3 and the Sp1/3 regulatory elements located in the first and second promoters, respectively (Canaff et al., 2008). The reason CaSR expression is enhanced by pro-inflammatory signals, thus leading to a reduction in serum calcium content, remains elusive. Some authors have speculated that this effect might counteract the increased bone resorption that is frequently observed in ill patients, in particular in patients suffering from rheumatoid arthritis and burn injury (Klein et al., 2016; Klein, 2018). In this context, pro-inflammatory cytokines have been shown to promote osteoclastogenesis and bone resorption, which lead to calcium release into the bloodstream (Walsh et al., 2005; Amarasekara et al., 2015); in turn, the augmented calcium level would eventually be rectified by the increased CaSR expression in the calcitropic tissues (Hendy and Canaff, 2016a; Klein et al., 2016).

A high level of extracellular calcium is commonly found at sites of inflammation during infections (Kaslick et al., 1970; Lin and Huang, 1991) and at sites of ischemic necrosis (Tzimas et al., 2004; Korff et al., 2006). Therefore, the calcium ion may function as a second messenger to promote inflammation by promoting chemokine/cytokine secretion and, thus, immune cell recruitment/activation. Indeed, Shi et al. (1996) found that extracellular calcium exerts a strong chemoattractant effect on macrophages, and Olszak et al. (2000) found that this chemokinetic effect is CaSR dependent. This implies that the CaSR is not only a responder of the inflammatory signal, but also a player in immune recruitment. Increasing numbers of studies have observed that the CaSR is expressed in immune cells where it mediates their activity.

### The calcium-sensing receptor in immune cells

One of the first evidences of a calcium-sensing mechanism inside immune cells that regulates their activity and cytokine production came from a study by Bornefalk et al. (1997), who found that extracellular calcium promoted both *in vitro* and *in vivo* the release of IL-6 from mononuclear blood cells. In the same year, the CaSR was found to be expressed in the hematopoietic precursor cells, in particular in mononuclear cells isolated from whole human and mouse bone marrow tissues (House et al., 1997). Afterwards, Yamaguchi et al. (1998) demonstrated that CaSR was indeed expressed in human peripheral blood monocytes. In monocytes, the CaSR was found to be functional and modulable, being able to mediate cell chemotaxis induced by extracellular calcium (Olszak et al., 2000). Two independent studies found that calcium was able to trigger IL-1β secretion from monocytes *via* the activation of the NLRP3 inflammasome, and demonstrated that this activation was dependent on CaSR expression and activity (Lee et al., 2012; Rossol et al., 2012). Following studies corroborated those findings, observing that the CaSR is able to trigger a pro-inflammatory response by activating the NLRP3 inflammasome in monocytes and differentiated macrophages (Liu et al., 2015; Jäger et al., 2020; Su et al., 2020) and to mediate constitutive micropinocytosis, thus contributing to antigen engulfment and subsequent antigen presentation (Canton et al., 2016).

Additionally, the CaSR was found to be expressed in other immune cells. In peripheral blood polymorphonuclear neutrophils, the CaSR regulates cell activation through the NF-κB pathway (Zhai et al., 2017). In lymphocytes, the CaSR has been seen to promote cytokine secretion through distinct pathways, including MAPKs and NF-κB, and to induce T-cell apoptosis by interacting with the transient receptor potential canonical (TRPC) 3 and TRPC4 channels through the PLC-IP_3_ pathway (Li et al., 2013; Wu et al., 2015b; Wu et al., 2015a; Zeng et al., 2016).

The CaSR is therefore a key player in immune cell activation and, as such, it seems to contribute to the pathophysiology of many inflammatory diseases, including myocardial infarction (Liu et al., 2015; Zeng et al., 2018), sepsis (Wu et al., 2015b; Wu et al., 2015a), rheumatoid arthritis (Jäger et al., 2020), and orchitis (Su et al., 2020).

### Autoimmunity and immune check-points inhibition

In addition to its active role in regulating immune response, the CaSR also seems to be an immune target in autoimmune reactions. As for idiopathic hypoparathyroidism and acute polyendocrine syndrome, CaSR-specific autoantibodies were identified in patient serum, implying a putative involvement of a CaSR-targeted autoimmunity in the etiology of those particular disorders (Li et al., 1996; Mahtab et al., 2017; Kemp et al., 2018). Mahtab et al. (2017) found specific CD8^+^ T cells able to react with CaSR peptides in the serum of patients suffering from idiopathic hypoparathyroidism, and Habibullah et al. identified particular subsets of CaSR-autoantibodies, which were able to target the CaSR and promote its activity, thereby exacerbating parathyroid gland inactivity (Habibullah et al., 2018).

Curiously, in several case studies of cancer patients receiving treatments for immune check point inhibitors, a well-established anticancer treatment that restores immune reactivity against cancer cells, the patients manifested hypocalcemia and hypoparathyroidism, which could be ascribed to the presence of CaSR-specific autoantibodies found in the sera (Piranavan et al., 2019; Trinh et al., 2019; Dadu et al., 2020; Lupi et al., 2020). The reason of this endocrine sequalae after the treatments with immune check-points inhibitors is still under investigation and yet not understood, but merit appropriate investigation to ameliorate long-term patients’ quality of life and guarantee their survival.

## Calcium-sensing receptor-driven tissue inflammation

Many studies agree on CaSR modulation of the inflammatory response by acting through the NLRP3 inflammasome and the NF-κB pathway (Figure 2). By doing so the CaSR mediates not only immune cells response, but also the inflammatory stimuli in different tissues, as described below.

### In the brain

Alzheimer’s disease (AD) is a neuro-dysfunction with huge social and economic impacts, especially in well-developed countries with advanced healthcare systems, where the elderly population is increasing. AD is the most recurrent form of dementia in elderly, characterized by cognitive impairment, caused by neurodegeneration and neuronal network loss (2022 Alzheimer’s disease facts and figures, 2022). The pathogenesis of AD is extremely complex and not yet completely understood, but partially ascribed to chronic neuroinflammation and the neurotoxic effects induced by the gradual deposition of interneuron plaques, formed of insoluble amyloid-β (Aβ) fibers, and by intraneuronal neurofibrillary tangles of hyperphosphorylated Tau proteins (Kumar et al., 2022). During the early phases of the disease, there is a progressive accumulation of toxic insoluble Aβ fibers due to the disruption of the metabolism of amyloid precursor protein (APP), an important factor in neuronal development (Dal Prà et al., 2019). In this context, the CaSR has been found to play a key role in the pathogenesis of AD and in modulating neuronal inflammation (Chiarini et al., 2016; Dal Prà et al., 2019). The first hint of a possible CaSR involvement in the pathogenesis of AD came from the discovery that soluble Aβ (sAβ) is able to bind the CaSR and to behave as a CaSR agonist (Ye et al., 1997; Conley et al., 2009; Dal Prà et al., 2014). Moreover, genetic CaSR polymorphisms have been seen to strongly associate with AD (Conley et al., 2009) and, in a particular animal model of AD, namely the 3xTg, the CaSR was found to be aberrantly up-regulated (Gardenal et al., 2017). sAβ-CaSR interaction seems to promote a noxious signaling in neurons, which leads to neuronal inflammation and death (Chiarini et al., 2016; Dal Prà et al., 2019). Indeed, sAβ-CaSR binding has been observed to promote the over-expression of new Aβ oligomers that progressively accumulate in neurons, causing subsequent cytotoxic effects that could be abolished through CaSR downregulation or with the administration of CaSR inhibitors (Ye et al., 1997; Conley et al., 2009; Armato et al., 2013; Bai et al., 2015; Chiarini et al., 2017; Feng et al., 2020). Moreover, sAβ-CaSR interaction has been shown to induce the synthesis of nitric oxide and vascular endothelial growth factor (VEGF)-A that concurrently participate in the AD pathogenesis (Dal Pra et al., 2005; Dal Prà et al., 2014).

Recent studies have reported a direct contribution of the CaSR in regulating the expression of pro-inflammatory cytokines in neurons such as IL-6, intercellular adhesion molecule-1 (ICAM-1), Regulated upon Activation normal T cell Expressed and presumably Secreted (RANTES) and monocyte chemotactic protein (MCP)-2 (Chiarini et al., 2020a). The CaSR was additionally found to mediate a pro-inflammatory stimulus after tissue damage, as observed in a mouse model of brain hemorrhage, where CaSR expression was up-regulated after injury, and its stimulation with Gd^3+^ increased NLRP3 and IL-1β expression (Wang et al., 2020).

Due to its direct involvement in neuroinflammation and AD dysfunction, the CaSR has been thought to behave as a danger-sensing/pattern recognition receptor in AD pathogenesis (Chiarini et al., 2020b) and, thus, it is thought to be a promising molecular target for AD treatment by using existing calcilytics (Chiarini et al., 2016; Dal Prà et al., 2019; Chiarini et al., 2020b).

### In the lungs

Pharmacological inhibition of the CaSR has recently been proposed as a new and effective therapeutic strategy for the treatment of asthma, given the putative pro-inflammatory role that the CaSR seems to play in the lungs (Yarova et al., 2015; Corrigan, 2020; Yarova et al., 2021). The first hint of a possible involvement of the CaSR in the pathophysiology of asthma came from the observation that in inflamed lungs of asthmatics there is an accumulation of known CaSR agonists, i.e., eosinophil cationic protein, spermine, spermidine, and putrescine, that seem to directly contribute to airway hyperresponsiveness (AHR) and lung inflammation (Kurosawa et al., 1992; Coyle et al., 1993; Gibson et al., 1998; Koller et al., 1999; Homma et al., 2005; Pégorier et al., 2006; North et al., 2013). In 2010, Cortijo et al. (2010) found that nickel ions were able to induce epithelial contraction and the release of pro-inflammatory cytokines by targeting and stimulating the CaSR, while CaSR inhibition through calcilytics abolished those effects. Afterwards, the proof of a direct involvement of the CaSR in the pathophysiology of asthma came from a study by Yarova et al. (2015), who observed that CaSR was over-expressed in bronchial smooth muscle cells of asthmatics and that pharmacological inhibition of the CaSR, by administering the calcilytic NPS2143 through inhalation, could abrogate AHR and lung inflammation. Later studies also confirmed the anti-inflammatory effects of calcilytics, which were found to be able to abolish the pro-inflammatory stimuli triggered by cigarette smoke extracts (Lee et al., 2017b) and by bacterial lipopolysaccharide (LPS) (Lee et al., 2017a). Recently, a panel of calcilytics, previously developed for the treatment of osteoporosis but found to be not clinically effective on bone anabolism, were tested in asthma models and resulted to be effective in counteracting asthmatic symptoms (Yarova et al., 2021). Thanks to their capacity to inhibit both AHR and lung inflammation, and thanks to their route of administration (i.e., inhalation, which can avoid systemic undesired effects), calcilytics are thought to be promising new therapeutics for the treatment of asthma, thus constituting a novel and effective replacement for existing steroids (Corrigan, 2020).

### In the cardiovascular system

The CaSR plays important roles in the physiology of the cardiovascular system. It is expressed and functional in cardiomyocytes, vascular smooth muscle cells (vSMCs), and vascular endothelial cells, where it regulates heart contractility, blood pressure, vessel tone (Wang et al., 2003; Schepelmann et al., 2015; Horinouchi et al., 2020), and even systemic mineral metabolism (Schepelmann et al., 2022). The CaSR is also implicated in the pathophysiology of cardiovascular diseases, such as myocardial infarction (MI), heart failure, hypertension, atherosclerosis, vascular calcification, and ischemia/reperfusion (I/R)-injury (Sundararaman and van der Vorst, 2021). Moreover, a CaSR polymorphism, A986S, has been associated with coronary artery diseases (März et al., 2007).

Cardiovascular morbidities are complex disfunctions, in which inflammation plays a major role in development and progression. Nonetheless, the CaSR seems to regulate the inflammatory response at the site of the vascular and cardiac lesions by directly acting both in tissue and in local immune cells. In a rat model of hypertension, in particular in vSMCs, the CaSR has been found to promote the release of pro-inflammatory cytokines through the NLRP3 inflammasome (Zhang et al., 2019). Leng et al. (2019) found that, in inflamed vascular endothelial cells, the CaSR promotes the release of the pro-inflammatory cytokines IL1-β and IL-18 through the activation of the NLRP3 inflammasome and the NF-κB pathway. In neonatal rat cardiomyocytes, pharmacological CaSR stimulation was found to enhance the LPS-driven pro-inflammatory stimulus, while CaSR inhibition attenuated the inflammatory response (Wang et al., 2013). Zhang et al. (2020) observed that, in a cardiac muscular cell line and in ventricular cardiomyocytes, the CaSR modulated monocyte chemotactic protein-1 (MCP-1)-driven apoptosis after I/R-injury. Conversely, a recent study from Guha et al. (2020) reported that, in inflamed aortic endothelial cells, CaSR stimulation by γ-glutamyl-valine reduced the expression of the pro-inflammatory cytokines IL-8, IL-6 and TNF-α. Nevertheless, most of the published studies outline the pro-inflammatory role of the CaSR in the cardiovascular system, as further evidenced by the fact that the CaSR has also been shown to promote cardiovascular lesions through the recruitment and activation of the immune cells (Sundararaman and van der Vorst, 2021).

Liu et al. (2015) observed that the CaSR, expressed in the macrophages, contributes to cardiac remodeling after MI *via* the activation of the NLRP3 inflammasome. Still under MI insult, the CaSR has been found to be up-regulated in peripheral and infiltrating neutrophils, and its pharmacological stimulation with calindol has been seen to promote neutrophil NLRP3 inflammatory response, furthermore provoking myocardial apoptosis and fibrosis (Ren et al., 2020). The CaSR has been found to mediate, *via* the NF-κB pathway, T lymphocyte activation and cytokine release in patients suffering from acute MI (Zeng et al., 2016) and to exacerbate cardiomyocyte lesions under myocardial ischemia and I/R-injury (Zeng et al., 2018).

In addition to its direct impact on the cardiovascular physiology and inflammation, the CaSR can putatively cause cardiovascular diseases by interfering with the metabolism of the adipose tissue and promoting adipose inflammation, which are typical hallmarks of obesity, a known risk factor for the development of cardiovascular diseases.

### In the adipose tissue

Among the many physiological roles that the CaSR plays, this receptor can also control lipid metabolism and adipogenesis (Bravo-Sagua et al., 2016; Mattar et al., 2020). In 2005, Cifuentes et al. (2005) found, for the first time, the CaSR to be expressed in adipose tissue. Afterwards, the CaSR was reported to regulate adipocyte differentiation and adipogenesis by promoting the expression of adipocyte regulatory transcription factors (He et al., 2012; Villarroel et al., 2013), to regulate preadipocyte proliferation through ERK1/2 activation (Rocha et al., 2015), and to inhibit lipolysis (Cifuentes and Rojas, 2008) by decreasing cAMP levels and inhibiting protein kinase A (PKA) activity (He et al., 2011). However, the CaSR has been shown to promote adipose inflammation, thereby altering lipid metabolism and possibly contributing to obesity (Villarroel et al., 2014; Bravo-Sagua et al., 2016). Adipose CaSR expression seems to be upregulated under obese-associated inflammatory stimuli (Cifuentes et al., 2010) and, on the other hand, pharmacological CaSR stimulation has been seen to promote the expression of the pro-inflammatory cytokines IL1-β, IL-6, TNF-α and the chemokine CCL2, through the NF-κB pathway (Cifuentes et al., 2012; Rocha et al., 2015) and the NLRP3 inflammasome (D’Espessailles et al., 2018).

Autophagy has also been shown to contribute to adipocyte dysfunction in obesity, and the CaSR seems to regulate this process by promoting the formation of the autophagosome (Mattar et al., 2018). The expression of the CaSR and autophagy markers were found to correlate with the percentage of body fat, thus implying a tight bond between the CaSR and autophagy in the pathophysiology of obesity (Mattar et al., 2020).

Obesity is also characterized by the infiltration of immune cells within the adipose tissue, which further contributes to adipose inflammation and lipid dysfunction, where the CaSR seems to be a key player in these processes. In THP-1 macrophages, CaSR stimulation with cinacalcet has been seen to activate the NLRP3 inflammasome and induce the expression of TNF-α and IL-1β in co-cultured preadipocytes (D’Espessailles et al., 2020). Moreover, a recent study from Thrum et al. showed that CaSR stimulation with increasing concentrations of extracellular Ca^2+^ promoted IL-1β expression, in particular in macrophages and adipocytes derived from obese patients, which were more sensitive to CaSR-induced pro-inflammatory stimulus compared to macrophages and adipocytes derived from healthy donors (Thrum et al., 2022).

Despite the numerous studies that attest a pro-inflammatory role, other recent reports have published contradicting results. Xing et al. observed that stimulating the CaSR with γ-glutamyl-valine abolished the TNF-α-induced pro-inflammatory stimulus in adipocytes (Xing et al., 2019); while Sundararaman et al. did not find any CaSR-dependent effects on visceral adipose inflammation in mice with adipose-specific CaSR knock-out (Sundararaman et al., 2021). In light of these contradictory findings, more studies are needed to clarify whether the CaSR induces inflammation in the adipose tissue and whether it does so by acting directly in the adipocytes or only by promoting immune cell infiltration and activity.

### In the digestive system

The CaSR seems to contribute to the inflammatory response in the upper digestive tract. In the epithelium of the esophagus, the CaSR seems to mediate a pro-inflammatory response through the activation of the NLRP3 inflammasome. This assertion is based on a recent observation, using *in vitro* and *in vivo* models of reflux esophagitis, where Tojapride, a natural formulation from Chinese traditional medicine, was seen to alleviate the inflammatory stimuli by interfering with the CaSR-NLRP3 signaling cascade (Yin et al., 2020). A very recent publication observed a pro-inflammatory role of the CaSR even in dental pulp cells, where it was found to mediate the expression of the pro-inflammatory markers IL-1β, IL-6, TNF-α and COX2-derived PGE_2_ under LPS challenge (An et al., 2022).

The CaSR is multifunctional in the intestine, where it mediates fluid absorption, gut motility, and the secretion of digestive hormones and electrolytes (Geibel et al., 2006; Wang et al., 2011; Tang et al., 2018). According to previous literature, the CaSR seems to exert a protective function against intestinal inflammation, which was extensively illustrated in a recent review from Iamartino et al. (2018). At the time, the authors reasoned that the CaSR, by acting as a nutraceutical sensor and, thus, by sensing byproducts of digestion, could link the beneficial effects of a healthy diet rich in calcium with intestinal health. Moreover, they speculated about how the CaSR could be a possible drug target using existing calcimimetics for the treatment of intestinal disorders, such as colorectal cancer and inflammatory bowel diseases (IBDs) (Iamartino et al., 2018). These authors based their hypotheses on previous reports that observed anti-inflammatory effects of the CaSR both *in vivo* and *in vitro*. Cheng et al. reported that intestine-specific CaSR KO mice were more susceptible to dextran sulphate sodium (DSS)-induced colitis. In this study, the animals lacking intestinal CaSR presented a higher recruitment of immune cells in the bowel, higher expression of pro-inflammatory cytokines, an altered composition of intestinal microbiome, a reduced expression of tight junction markers, e.g., claudin 2, and, thus, a compromised epithelial integrity (Cheng et al., 2014). These findings were corroborated by subsequent studies where CaSR stimulation with either poly-L-lysine, glutamyl dipeptides, or l-amino acids reduced inflammation *in vitro*, i.e., in TNF-α challenged colorectal cancer cell lines Caco2 and HT29, and *in vivo* both in DSS-induced colitis mouse models (Mine and Zhang, 2015a; Mine and Zhang, 2015b; Zhang et al., 2015) and in LPS-challenged piglets (Liu et al., 2018). Recent studies further observed an anti-inflammatory effect of the CaSR, showing how CaSR stimulation with either tryptophan or spermine can enhance intestinal barrier integrity and the expression of tight junction markers, while also attenuating the pro-inflammatory stimuli driven by TNF-α, enterotoxigenic *E. Coli*, or LPS in porcine intestinal epithelial cells (Liu et al., 2021a; Liu et al., 2021b; Gao et al., 2021). Moreover, tryptophan-driven CaSR stimulation was seen to increase the production of endogenous defensins, hence improving host immune defense (Gao et al., 2021).

Nevertheless, new studies reported conflicting data regarding the effects of the CaSR in bowel inflammation, outlining a pro-inflammatory impact. In the attempt to assess the therapeutic applicability of the calcimimetics for the treatment of IBDs, Elajnaf et al. administered by gavage either two positive allosteric CaSR modulators, cinacalcet and a newly synthetized not absorbable calcimimetic, GSK3004744 (Sparks et al., 2017), or calcilytic NPS2143, to DSS-treated mice, which were used as a preclinical model of colitis. These authors found that pharmacological stimulation of the CaSR with cinacalcet increased the serum levels of TNF-α, IL-6, and IL-1α, implying a systemic effect of cinacalcet in enhancing inflammation, while the effects of GSK3004744, which acted locally and exclusively within the mucosa, were negligible. Surprisingly, the administration of NPS2143 reduced distress scores and immune cell infiltration in the mucosa. The authors reasoned that those effects were most likely systemic, acting on circulating immune cells, since the gut-restricted GSK3004744 did not significantly influence the inflammatory parameters (Elajnaf et al., 2019). In a subsequent study from Iamartino et al. (2020), it was observed a direct pro-inflammatory effect of the CaSR in intestinal epithelial cells. Using two colorectal cancer cell lines over-expressing the CaSR fused to the green fluorescent protein (GFP), HT29^CaSR-GFP^ and Caco2^CaSR-GFP^ cells, and comparing them with their corresponding negative controls (HT29^GFP^ and Caco2^GFP^), they observed that only those expressing the CaSR and treated with the positive allosteric CaSR modulator, NPS R-568, had an increased expression of pro-inflammatory markers IL-8, IL-23α, IL-1α, CSF1, CCL20, COX2, and PDL1, and increased secretion of IL-8 (Iamartino et al., 2020). These findings were further confirmed by a recent study from the same research group, where they compared the CaSR-specific R-enantiomers with the CaSR-unspecific S-enantiomers derived from the allosteric CaSR modulators NPS R-568 and NPS2143. According to the authors this was done in order to exclude possible off-target effects that the allosteric CaSR modulators (the R-enantiomers) may have exerted on inflammatory markers in a CaSR-independent manner, possibly acting on other GPCRs or calcium channels. It was observed that only R-enantiomers were able to modulate intracellular calcium mobilization and IL-8 secretion, thus demonstrating a CaSR-dependence of the pro-inflammatory effects induced by the calcimimetic NPS R-568 (Schepelmann et al., 2021).

One of the possible explanations for the discrepancy of the published results could be the type of molecules used to stimulate the CaSR and the cell models employed. Despite targeting the CaSR with less-specific nutraceutical molecules, such as polyamines, dipeptides and amino acids, Elajnaf et al. (2019) were the first to test highly CaSR-specific synthetic compounds in animal models of colitis. Moreover, Iamartino et al. (2020) and Schepelmann et al. (2021) used colorectal cancer cell lines over-expressing the CaSR compared to not transduced cells (Mine and Zhang, 2015a; Mine and Zhang, 2015b; Zhang et al., 2015; Liu et al., 2018), where, in particular in the Caco2 and HT29 cell lines, the endogenous CaSR expression is reduced or even undetectable.

Therefore, due to the conflicting data, the role of the CaSR in intestinal inflammation is still unresolved and thus needs further clarification. This is important because intestinal CaSR may be linked to the gastrointestinal side effects observed in patients under prolonged treatment with cinacalcet or etelcalcetide, the newly FDA-approved calcimimetic. Pharmacovigilance studies have reported that cinacalcet and etelcalcetide are not well-tolerated after prolonged periods of administration, since patients manifest distress and discomfort, suffering from nausea, vomiting and gastrointestinal bleeding (Block et al., 2017; AMGEN Sensipar, 2022). Whether these effects are connected to a possible pro-inflammatory role of the CaSR in the intestine is still under debate and necessitates clarification, in particular in light of the possible detrimental effects that a calcimimetic-based therapy could exert in patients suffering from colitis. Moreover, clarifying whether pharmacological CaSR inhibition has beneficial effects against intestinal inflammation will open the way to new therapeutic approaches based on the use of calcilytics for the treatment of IBDs.

### In the kidneys

It is well known that the CaSR regulates mineral balance in the kidneys, but it also seems to be involved in the pathophysiology of several renal defects.

These include I/R renal injury (Weekers et al., 2015), further exacerbated by diabetic comorbidity, where the CaSR seems to enhance oxidative stress and boost the inflammatory response (Hu et al., 2018).

Tubular interstitial defects, where the CaSR, expressed in the inner medullary collecting duct cells, seems to induce tubular interstitial fibrosis by promoting collagen expression under the pro-inflammatory stimulus driven by IL1-β (Wu et al., 2019).

In the medullary thick ascending limb, the CaSR seems able to mediate sodium excretion by promoting COX-2 expression and prostaglandin production, in particular PGE_2_, and it does so by inducing the expression of TNF-α, linking in this way a pro-inflammatory stimulus to natriuresis (Wang et al., 2001; Wang et al., 2002; Ferreri et al., 2012).

The CaSR has been hypothesized to provoke glomerular damage by coupling with the TRPC6 and boosting intracellular calcium influx, which has been ascribed as one of the principal culprits in the pathophysiology of membranous nephropathy (Huang et al., 2021) and in acute kidney injuries that frequently occur in septic patients (Yadav et al., 2021). However, as the authors suggested, direct evidences on these regards are missing and, thus, further studies are needed to clarify the possible implication of the CaSR in glomerular defects under inflammatory stimuli.

Finally, the CaSR has also been associated to nephrocalcinosis and nephrolithiasis, common inflammatory renal defects, which are also recurrent morbidities in ADH patients and are strongly associated with hypercalciuria (Roszko et al., 2022). Moreover, single nucleotides CaSR polymorphisms have been associated to calcium nephrolithiasis, in particular the rs6776158 (A > G) polymorphism, resident on CaSR first promoter, which is associated with decreased transcriptional activity (Vezzoli et al., 2013; Vezzoli et al., 2019). However, the underlying molecular mechanisms that link CaSR genetic variability to kidney stones are still uncovered.

### In other tissues

Because hypercalcemia is a well-known risk factor for pancreatitis (Felderbauer et al., 2007), and given the tight bond between the CaSR and serum calcium homeostasis, it was speculated that CaSR mutations or SNPs could be linked to pancreas inflammation. However, there are conflicting results regarding this topic and thus the question is still under debate (Felderbauer et al., 2007; Murugaian et al., 2008; Stepanchick et al., 2010; Xiao et al., 2017; Ewers et al., 2021).

In the bones, the CaSR is known to promote osteogenesis and mineralization (Dvorak-Ewell et al., 2011; Chang et al., 2013), but it has also been seen to promote aberrant new bone formation in ankylosing spondylitis (AS), a chronic inflammation of the axial skeleton. The CaSR was found to be aberrantly up-regulated in osteoblasts derived from patients suffering from AS. Moreover, in several animal models of AS, CaSR inhibition attenuated AS symptoms (Li et al., 2020). Despite the pivotal calcitropic role of the bone tissue, where the CaSR is markedly expressed, reports addressing a direct function of the CaSR in bone inflammation are still missing.

Interestingly, metastatic breast cancer cells seem to exploit CaSR capability to drive cytokine secretion, in order to promote their chemotaxis and angiogenesis, as observed in the highly invasive MDA-MB-231 breast cancer cell line stimulated with CaSR allosteric modulator NPS-R568 (Hernández-Bedolla et al., 2015; Hernández-Bedolla et al., 2016). Furthermore, in MDA-MB-231 and in the MCF-7 breast cancer cell lines, CaSR stimulation was found to enhance the expression of Rab27B, a Rab GTPase that is involved in endosomal trafficking (Zhen and Stenmark, 2015), promoting in this way the secretion of pro-inflammatory cytokines and chemokines such as IL-6, IL-1β, IL-8, IP-10 and RANTES (Zavala-Barrera et al., 2021).

## Ca^2+^-vitamin D-calcium-sensing receptor asset in viral infection and COVID-19 pathology

Ca^2+^ is involved in all steps of the viral life-cycle, including virion fusion into the host cell, viral protein synthesis, viral maturation and release. Viruses, including coronaviruses and the SARS-CoV family, alter calcium concentrations within cells, promoting calcium influx into the cytoplasm (Zhou et al., 2009; Millet and Whittaker, 2018). Viral envelope protein E is up-regulated during infection and functions as a calcium channel that induces calcium influx inside the cell, which in turn facilitates viral-host interaction and fusion. Moreover, the rise of intracellular calcium causes the activation of the NLRP3 inflammasome and its down-stream pro-inflammatory signaling, leading to systemic comorbidities (Nieto-Torres et al., 2014; Nieto-Torres et al., 2015). Therefore, calcium channel blockers have been tested and found to be effective against viral infections, as seen for influenza A virus, Japanese encephalitis virus, hemorrhagic fever arenavirus and ebolavirus (Berlansky et al., 2022). Nonetheless, SARS-CoV-2, which is responsible of the current COVID-19 pandemic, alters calcium homeostasis to favor its virulence by boosting intracellular calcium influx. Therefore, calcium channel blockers are under investigation as therapeutics against this novel world-wide diffused infection (Alsagaff et al., 2021; Berlansky et al., 2022).

Whether correlated or not to the viral need of calcium ions for the infective processes, many epidemiological studies have reported a high incidence of hypocalcemia in COVID-19 patients, especially in those hospitalized (with an incidence of up to 80%) and admitted to intensive care units. Importantly, a low calcium level appears to be an unfavorable prognosis marker, as observed in patients with lower serum calcium who manifest more severe symptoms and are more likely to be in need of intensive care treatment, compared to those with mild or normal calcemia (Cappellini et al., 2020; Zhou et al., 2020; Alemzadeh et al., 2021; di Filippo et al., 2021; Torres et al., 2021).

Correlated to calcium homeostasis, COVID-19 patients also manifest an alteration of vitamin D and phosphate metabolism, thus implying a systemic derangement of minerals homeostasis. Hypovitaminosis D has frequently been diagnosed in COVID-19 patients (Bennouar et al., 2021; Elham et al., 2021), and its low level has been associated with poor prognosis, most likely due to the loss of the immune regulatory role played by its active metabolite, 1,25-dihydroxyvitamin D3 (Bassatne et al., 2021; Lisco et al., 2021).

An epidemiological study conducted by Yang et al. observed that hypophosphatemia was also frequent in COVID-19 patients and its low level was seen to correlate with the severity of the infection (Yang et al., 2021).

Numerous studies have reported the occurrence of multiple endocrine sequalae post SARS-CoV-2 infection, affecting gonads, bones, hypothalamus, pituitary, thyroid, and adrenal glands (Lisco et al., 2021). Therefore, it is plausible to include parathyroid defects among the endocrine complications in COVID-19 infection, which would explain, at least in part, the inappropriately low levels of calcium, vitamin D, and phosphate found in infected patients (Lisco et al., 2021). A first hint about this perspective comes from a recent cross-sectional study, where PTH levels were found to be low in roughly 40% of COVID-19 patients with hypocalcemia, hence implying a defect in parathyroid functionality (Hashemipour et al., 2022).

PTH decrease and the decline in serum calcium are frequently found in critically ill patients and, as aforementioned, the CaSR is putatively addressed as possibly responsible for these defects. Because viral infection induces inflammatory stimuli, as for the activation of the NLPR3 inflammasome, the pro-inflammatory cytokines could possibly up-regulate parathyroid CaSR expression, as seen for the NLRP3-associated IL1-β cytokine. Based on this assumption, viral-induced CaSR over-expression would render parathyroid glands hypofunctional, causing PTH impairment and hypocalcemia; nevertheless, studies on this regard are still missing.

Another piece of evidence that supports a putative involvement of the CaSR in the infective SARS-CoV-2 processes is the fact that CaSR activation augments intracellular calcium levels by inducing the release of calcium from the endoplasmic reticulum and promoting calcium influx from the extracellular environment through the activation of the store operated calcium channels and the transient receptor potential (TRP) channels (Xie et al., 2017; Hannan et al., 2018; Onopiuk et al., 2020). Therefore, CaSR capability to increase intracellular calcium could support viral infective mechanisms, although direct evidences are missing and merit investigation.

In addition, upon viral infection, the CaSR could also be upregulated in immune cells and eventually may exacerbate the inflammatory response in severely ill COVID-19 patients.

Nevertheless, these assumptions are mere speculations due to the lack of CaSR-focused studies in SARS-CoV-2 infected patients. To date, it is not possible to explain the etiology behind hypocalcemia during COVID-19 infection, but parathyroid defects that are yet uncovered are highly expectable and a CaSR aberrant over-activity is plausible; therefore, additional investigations into these questions are needed.

## Conclusion

The CaSR is a multifunctional ubiquitously expressed receptor important for many physiological processes. This functional variability is also reflected by the yin-yang role it plays in the pathophysiology of many diseases, including cancer and inflammation. Despite some exceptions, accumulating studies are outlining how the CaSR induces pro-inflammatory stimuli, as summarized in Table 1, and therefore it is an important factor in the pathophysiology of many inflammatory diseases. The CaSR can modulate the inflammatory response either by acting directly within the tissues or by modulating immune cell activation and motility. For this reason, pharmacological CaSR inhibition with specific calcilytics has gained interest in recent years as a possible treatment and mitigation of aberrant inflammatory stimuli, which are usually observed in diseases such as asthma, Alzheimer’s disease, obesity, and inflammatory bowel diseases; and, by extension, for the treatment and/or management of sepsis, burn injuries and rheumatoid arthritis.

**TABLE 1:** CaSR-driven pro-inflammatory signals in different tissues and cell types.

Effects	Ligand	Pathway	Cell type/tissue	References
Chemotaxis induction	Ca^2+^, spermine, NPS R-467	n/a	Monocytes	Olszak et al. (2000)
IL-1β secretion/immune activation	Ca^2+^, Gd^3+^, NPS R-568	NLRP3 inflammasome	Monocytes/macrophages	Lee et al. (2012), Rossol et al. (2012), Liu et al. (2015), D’Espessailles et al. (2020), Jäger et al. (2020), Thrum et al. (2022)
Constitutive micropinocytosis	Ca^2+^	NLRP3 inflammasome	Macrophages	Canton et al. (2016)
IL-6 and myeloperoxidase secretion/immune activation	Cinacalcet, Calindol vs. CaSR inhibition with Calhex 231	NF-κB, NRP3 inflammasome	Neutrophils	Zhai et al. (2017), Ren et al. (2020)
Cytokines secretion	Ca^2+^, Gd^3+^, NPS R-568	MAPKs and NF-κB	Lymphocytes	Li et al. (2013); Wu et al. (2015a), Zeng et al. (2016)
Induction of AD culprits (i.e., Aβ synthesis, nitric oxide and VEGF-A)	sAβ, Ca^2+^ vs. CaSR inhibition with calcilytics	cPLA2/PGE2	Neurons and cortical astrocytes	Ye et al. (1997), Dal Pra et al. (2005), Armato et al. (2013), Dal Prà et al. (2014), Bai et al. (2015), Chiarini et al. (2017), Feng et al. (2020)
Cytokines secretion	sAβ, Gd^3+^	CaMKII/NLRP3 inflammasome	Neurons	Chiarini et al. (2020a), Wang et al. (2020)
Cytokines secretion/airway hyperresponsiveness	Ca^2+^, nickel, polycations,	MAPKs, Ca^2+^_int_ mobilization, cAMP breakdown	Airway epithelial cells/lungs	Cortijo et al. (2010), Yarova et al. (2015)
Inhibition of inflammation and airway hyperresponsiveness	Calcilytics	n/a	Lungs	Yarova et al. (2015), Lee et al. (2017a), Lee et al. (2017b), Yarova et al. (2021)
Cytokines secretion	Proof by CaSR inhibition with calcilytics	NLRP3 inflammasome	Vascular smooth muscle cells	Zhang et al. (2019)
IL1-β and IL-18 secretion	Proof by CaSR inhibition with NPS2143	NLRP3 inflammasome and NF-κB	Vascular endothelial cells	Leng et al. (2019)
TNF-α and IL-6 secretion	Gd^3+^ vs. CaSR inhibition with NPS2390	n/a	Cardiomyocytes	Wang et al. (2013)
Cytokines secretion	Cinacalcet, Gd^3+^, spermine	NLRP3 inflammasome and NF-κB	Adipocytes	Cifuentes et al. (2012), Rocha et al. (2015), D’Espessailles et al. (2018)
Bile acid-driven inflammation	CaSR and NLRP3 interference with Tojapride	NLRP3 inflammasome	Esophagus	Yin et al. (2020)
Cytokines expression	NPS R-568 vs. CaSR inhibition with Calhex 231	PI3K-AKT	Dental pulp	An et al. (2022)
Anti-inflammatory effects and host immune defense	poly-L-lysine, glutamyl dipeptides, l-amino acids, tryptophan, spermine	n/a	Colon and colorectal cancer cell lines	Mine and Zhang (2015a), Mine and Zhang (2015b), Zhang et al. (2015), Gao et al. (2021), Liu et al. (2018), Liu et al. (2021a), Liu et al. (2021b)
Cytokines secretion and colitis exacerbation	Cinacalcet, NPS R-568 vs. CaSR inhibition with NPS2143	n/a	Colon and colorectal cancer cell lines	Elajnaf et al. (2019), Iamartino et al. (2020)
Renal I/R injury	NPS R-568 vs. CaSR inhibition with NPS2143	n/a	Kidneys	Weekers et al. (2015), Hu et al. (2018)
Expression of collagen I and III (tubulointerstitial fibrosis)	Proof by CaSR inhibition with Calhex 231 and NPS2143	n/a	Inner medullary collecting duct cells	Wu et al. (2019)
Cytokines secretion	NPS R-568	n/a	Breast cancer cell lines	Hernández-Bedolla et al. (2015), Hernández-Bedolla et al. (2016), Zavala-Barrera et al. (2021)

Because the CaSR is ubiquitously expressed, pharmacological CaSR targeting exerts systemic and often unwanted effects, as for example observed in patients treated with cinacalcet or etelcalcetide, which manifest gastrointestinal distress. Therefore, new routes of administration and new strategies for tissue-specific delivery of CaSR-molecules are required to avoid undesired and possible detrimental systemic effects.

Lastly, given the possible involvement of the CaSR in the pathogenesis of COVID-19 infection, it is tempting to speculate about a possible applicability of calcilytics as effective therapeutics to counteract SARS-CoV-2 infection or, at least, to mitigate hypocalcemia, reducing patients’ morbidities. However, these assumptions need to be extensively investigated in future studies.
